# 3D Printing of Bone Substitutes Based on Vat Photopolymerization Processes: A Systematic Review

**DOI:** 10.1155/2023/3901448

**Published:** 2023-04-08

**Authors:** Simon Enbergs, Jacob Spinnen, Tilo Dehne, Michael Sittinger

**Affiliations:** Tissue Engineering Laboratory, BIH Center of Regenerative Therapies, Department of Rheumatology & Clinical Immunology, Charité-Universitätsmedizin Berlin, Charitéplatz 1, Berlin 10117, Germany

## Abstract

Treatment options for critically sized bone defects are currently limited to metal osteosynthesis, autologous bone grafting, or calcium-based implants to bridge the gap. Additive manufacturing techniques pose a possible alternative. The light-basedthree-dimensional printing process of vat photopolymerization (VP) is of particular interest since it enables the printing of complex scaffold architectures at high resolution. This review compares multiple vat photopolymerization processes as well as the employed resin components' interactions with musculoskeletal cells and tissue. The results show an outstanding printing capability, exceeding the potential of other printing methods. However, despite the availability of various biocompatible materials, neither the mechanical strength of bone nor the scale necessary for clinical application has been achieved so far when relying on single material constructs. One possible solution is the development of adaptive hybrid constructs produced with multimaterial VP.

## 1. Introduction

Clinical treatment of bone defects caused by trauma, diseases, or past surgery is a fundamental part of modern patient care. If a patient's endogenous healing process is insufficient for load-bearing usage and a noninvasive therapy option is no longer suitable, bone grafts or implants are often inserted during surgical reconstruction to regain physiological function [1]. Especially in the case of nonunion fractures and major bone loss due to invasive tumors, surgical intervention and implementation of material to reestablish a continuous bone structure are required [2].

Currently, autografts are considered the gold standard for the reconstruction of large bone defects. With a share of 55% (54,000 patients), it was the most frequently used method in orthopedic implant surgery in Germany in 2018 [3, 4]. Their widespread use is due to several factors: autologous bone grafts exhibit osteogenic, osteoinductive, and osteoconductive properties, leading to an average fracture reunion rate of 91%, while simultaneously not eliciting an immune response or carrying any risk of transmitting infectious diseases [5, 6]. Harvest and reimplantation take place within a single operative procedure, thus negating the need for storage and the costs associated therewith. The donor site depends on the area of application: in orthopedic operations, the iliac crest and the tibia are used for spinal fusions and tubular bone treatments [7]. In the reconstruction of the mandible, the fibula is applied as the replacement of the damaged bone [8]. The main disadvantage of this procedure is the inherent donor site morbidity. Autologous bone grafting carries an 8.9% incident rate of major complications, including permanent nerve damage, chronic pain, loss of physiological function, extensive blood loss, and need for reoperation. An additional 20.9% leads to minor complications occurring at the donor site, encompassing superficial wound infections and impermanent loss of sensation [9, 10].

These complications at the harvest site could be circumvented using bone substitute materials, allowing for optimal adaptation to the patient and accelerated healing. In several clinical interventions, such materials are currently in use: bone void filling in various defects, long-bone fracture treatment, and spinal fusions [11]. These substitutes can be produced in multiple ways.

Additive manufacturing (AM) can produce bone substitutes that suit clinical requirements adequately by adjusting the properties of the implant to the specific defect. Such a patient-specific implant would be designed by analyzing the radiologic image and consequently creating a computer-assisted design (CAD) of the bone construct. With a few exceptions, additive manufacturing methods are based on a layer-by-layer approach, which consists of printing successive two-dimensional layers formed on top of each other [12, 13].

There are various additive manufacturing techniques suitable for fabricating medical implants. These are categorized and defined by the international organization for standardization (ISO/ASTM 52900:2015). One major group of material-deposition printing systems such as fused filament fabrication (FFF) and extrusion-based printers, as well as inkjet-basedthree-dimensional (3D) printers, stack externally stored materials directly upon each other. The temperature difference between the print head and its surroundings is an essential factor in this method since a thermoplastic polymer filament needs to be heated to reach its melting point and then solidify via cooling to room temperature after printing. The other main group of printing processes relies on the process of chemical bonding. This category also includes vat photopolymerization (VP) methods. The first patented VP technique, stereolithographic addition, was developed by Chuck Hull in 1986 and has evolved over the years [14]. VP is a group of additive manufacturing techniques that uses light to initiate a chain reaction on a layer of photosensitive resin (ISO/ASTM 52900:2015). Extrusion-based printers rely on the physical movement of the printhead, limiting their resolution to the slack in its guiding equipment. VP methods, however, can achieve a high resolution by using an optical system, increasing the *x*-*y* resolution to the *µ*m wide spot size of the laser beam used for polymerization, which enables the creation of task-adjusted structures, allowing the translation of anatomical and functional aspects to a patient-specific scaffold [15].

In contrast to other printing technologies, VP has particular benefits of relevance for the production of bone substitutes. Photopolymerization offers the capability to print not only hydrogels but also mechanically stiffer polymers such as poly (ethylene glycol) diacrylate (PEGDA) or methacrylated poly(D,L-lactide-co-*ε*-caprolactone) (PLCL) without the heat of an FFF printer [16]. Thus, heat-unstable bioactive substrates can be added directly to the printing process. At the same time, VP enables the addition of ceramic particles to the structure, similar to extrusion-based printing technologies. However, the size of the particles is limited to the layer height and the viscosity of the resin. The photopolymerization of particle-containing resins leads to “surface enrichment.” This entropy-based effect allows the particles to appear on the surface without being coated by a polymer layer [17]. Dienel et al. and Guillaume et al. have applied this surface enrichment phenomenon to stimulate new bone formation by adding beta-TCP or hydroxyapatite particles [18, 19].

3D manufactured bone substitutes require several properties to be clinically viable [20]: (1) the scaffolds architecture must include either an interconnected porosity and/or sufficient biodegradability to allow for an appropriate ingrowth of blood vessels and cell migration of osteoblasts and osteoclasts. (2) The material used needs to be biocompatible without eliciting an immunological response. (3) Furthermore, it must possess mechanical properties surpassing or equaling the physiological requirements at the defect site. This mechanical strength in the range of 100 to 200 MPa poses a fundamental problem in the development of bone constructs [21]. Unlike in other tissue constructs, the balance between a cell-supportive osteoconductive environment and the required resilience to compressive and elastic forces is harder to achieve in bone scaffolds due to the negative feedback of an increasing polymer/material density on cell viability. The rising density increases the mechanical strength as well as the diffusion resistance, resulting in a lack of transport of nutrients and waste products. The range in which both adequate cellular viability and sufficient mechanical stiffness are attained has previously been described as the so-called “biofabrication window” [22, 23]. To meet both requirements simultaneously, the resins and polymerization techniques have to be adjusted. In the majority of cases, these processes employ only one material composition as the printing substrate. Thus, the obtained results are mainly attributed to the properties of the selected materials. This is in contrast to multimaterial scaffolds, in which the characteristics of the materials can be chosen according to the different tasks, creating a hybrid scaffold. A novel generation of light polymerizing printers utilizes multiple vats containing resins with differing attributes [24].

To understand the benefits and drawbacks of both new and old VP methods, this review provides an overview of the main VP methods and the materials used in research. It also aims to evaluate and discuss their clinical applicability and the perspective of VP in bone substitute manufacturing.

## 2. Methods

The studies and articles used in this review were found by a systematic search in the MEDLINE digital database (Pubmed). The search was performed using MeSH terms (Pubmed). Keywords included vat photopolymerization; stereolithography; digital light processing; two-photon polymerization; and bone. The keywords were used for the search either in combination with Boolean operations (AND, OR, NOT) or by themselves. The search mechanism automatically included searches for synonyms and related words.

A total of 412 literature references were found. Further screening, with the criteria listed in Table 1, included a total of 50 papers. The references in the papers were also taken into consideration.

According to the inclusion criteria, the research area had to be related to VP as well as bone scaffold development. There were no restrictions on whether in vitro or in vivo studies were conducted. Publications with human as well as animal cells were included. However, if no assessment of the biological interactions between the scaffold and cells was described, the publication was excluded. Exclusion criteria dismissed papers if the full text was unavailable in English and published before 2010/after September 2022.

## 3. Vat Photopolymerization Techniques and Materials

### 3.1. Methods of Vat Photopolymerization

**Figure 1:**
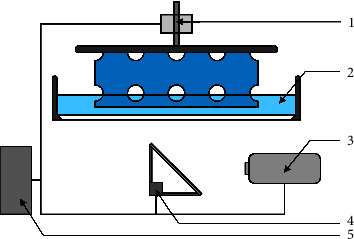
VP setup; 1: actuator; 2: resin-filled vat, 3: light source; 4: optical system; 5: controlling system

A typical VP setup usually consists of four different components: (1) the vat, filled with photosensitive resin; (2) a light source coupled with an optical system that either directs the light or limits light exposure; (3) a controlling system, which stores and distributes the operation steps; and (4) a physical actuator to move the build plate (Figure 1). The light intensity must exceed a resin-specific threshold for polymerization to occur. The controlling system regulates the energy accumulation within the resin by adjusting the optical system that guides the polymerization in the vat and enables the creation of 3D structures. However, several different VP methods can be distinguished from each other. Generally speaking, all of the systems listed in Table 2 are capable of printing constructs that appear to be suitable as bone substitutes. However, the different printing methods vary in how they affect several construct properties. A comprehensive overview of the features and limitations of each method is given in Table 2 and the following paragraphs.

#### 3.1.1. Stereolithography (SLA)

**Figure 2:**
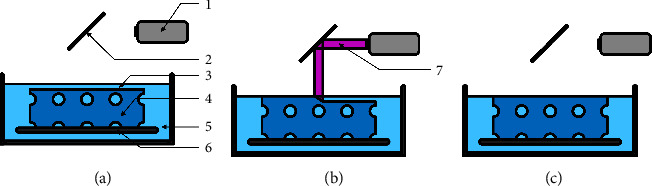
Schematic manufacturing process with bottom-up SLA printers, which place the laser source above the vat, and the construct is built facing up. (a) End of a previous layer after movement of the print platform for the next layer; (b) printing process of the next layer; (c) finishing of the new layer; (1: laser; 2: mirror (*x*/y axis); 3: layer height; 4: scaffold; 5: resin; 6: print plate; 7: laser beam.

SLA is the first generation of VP-based 3D printing. The operating principle is the tracing of a focused laser beam that is oriented over and drawn across a vat in a preprogrammed pattern, crosslinking any photosensitive resin that is illuminated. The laser source emits only light of a specific wavelength, which is deflected by an adjustable mirror and directed to scan along the planned path. The spot size at the focal point of the laser beam defines the minimal feature size, while the precise motion of the mirror enables the high x-y accuracy/resolution. Once a layer is completely cured, the printing platform moves slightly by a previously defined height, establishing the layer height. A new resin then flows over the cured polymer and covers it. This movement of the printing platform can either start just below the surface of the resin and move down further into the vat (bottom-up SLA) or start a layer height above the bottom of the vat and travel up through the resin-filled vat (top-down SLA) [32]. While both methods are commonly used, they both have their drawbacks. Bottom-up SLA begins at the very top of the resin surface and moves downwards (Figure 2). The distance between the surface and the platform is defined by the preset layer thickness. To ensure a consistent layer thickness, the surface of the resin has to be smooth. Therefore, the material's viscosity must be chosen carefully, and the printing time needs to be prolonged to facilitate the liquid settling down and to recoat the printed construct. Especially in the case of bone substitute manufacturing, this viscosity restraint negatively impacts the result. Since the viscosity increases with higher polymer concentrations, the viscosity restriction directly affects the polymer density and thus the mechanical attributes of the construct. Another disadvantage of the bottom-up procedure is the increased amount of resin required since the construct height is restricted by the maximum vat depth to allow the new resin to reach the top layer. This is especially relevant if cells are implemented into the construct, due to an increase in the amount of excess cell material wasted in the surrounding resin. However, this surrounding resin is a key advantage of the bottom-up approach via the structural support for the printed object, facilitating the printing of wider overhangs without additional support structures. In the case of bone substitutes, this benefit is less relevant due to the sufficient structural integrity of the printed construct.

**Figure 3:**
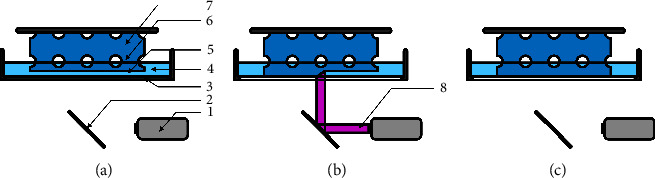
Schematic manufacturing process with top-down SLA printers, which place the laser source under the resin vat. The construct is built facing upside down. (a) End of a previous layer after movement of the print platform for the next layer; (b) printing process of next layer; (c) finishing of new layer; 1: laser; 2: mirror (*x*/y axis); 3: glass plate; 4: resin; 5: layer height; 6: scaffold; 7: print plate; 8: laser beam.

Top-down SLA printers (Figure 3) function the other way around, and light cures the resin through a light-permissive window at the base of the vat. After the completion of a layer, a sequence of steps prepares the printer for the next cycle. First, the polymerized resin detaches by tilting the base of the vat. Subsequently, the building plate, which now acts as an upper boundary, raises, allowing the new resin to fill the void space. After a set amount of time is needed for the resin to flow, the print plate lowers again to the defined layer height over the base [33]. Thus, the level of resin can be kept lower, increasing the ratio of polymerized to remaining resin. In the case of cell-laden resins, this leads to a decrease in price and print preparation work. Since the photopolymerization is not performed on the surface, inhibition of the photoinitiator activity caused by oxygen can be avoided, which facilitates sufficient polymerization. Furthermore, advantages of the bottom-up approach include that the illuminated surface is always smooth, thereby facilitating improved print quality with highly viscous resins. One disadvantage is that the detachment can fail if the printed object adheres to the window, leading to print failures. In contrast to the bottom-up method, the top-down technique requires both external as well as internal support material in the case of architectural designs with overhangs due to the lack of a support bath. This affects the possible architecture of the bone substitute and increases the extent of postprint processing.

#### 3.1.2. Digital Light Processing (DLP)

**Figure 4:**
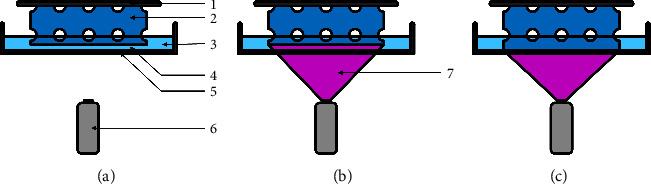
Schematic manufacturing process with a DLP system. (a) End of a previous layer after movement of the print platform for the next layer; (b) projection of new layer; (c) finishing of new layer; 1: print platform; 2: scaffold; 3: resin. 4: layer hight; 5: glass plate; 6: projector; 7: projection.

Another variation of VP uses DLP technology which is projection-based and differs from the laser-based scanning SLA printer in the light source. A projector illuminates the entire layer at once, initiating the polymerization after surpassing the photoinitiations specific energy threshold (Figure 4). For this purpose, 3D models are sectioned into thin slices/layers and represented by a series of images. Within this projecting system, structures are printed by the sequential projection of each image into a vat of resin as the printing plane moves away after each layer. Curing a whole layer before the printing plane moves upwards allowing for the resin to fill the gap between the object and the window. This procedure is repeated with each additional layer, as shown in Figure 4. In terms of speed, the ability to polymerize an entire layer at once is a considerable improvement over the aforementioned techniques. A crucial aspect of the accuracy is determined by the maximum resolution of the pixel-based projecting system. The size of the pixel on the resin defines the minimal feature size. However, the maximal *x*-*y* resolution is lower since the finest possible adjustment in the plane is the illumination in the adjacent pixel, leading to jagged diagonal lines. This problem is addressed by projection-micro-stereolithography (P*µ*SL), which has increased the maximum resolution by focusing the projected light through a lens system, concentrating the entire projection onto the printing plane [34, 35]. A by-product of the process is the constraining of the print size by the same scaling factor. To overcome this obstacle, a mirror is integrated into the system to redirect the focused projection across the printing area, leading to an increase in size [36].

The DLP method is frequently used in the development of bone substitutes. In particular, this process is used when ceramic particles are added to the resin to increase the mechanical strength of the constructs, as further elaborated in chapter 3.2. Even though the maximal resolution is smaller compared to the laser-based SLA method, its accuracy is enough for sufficient porosity within the construct and bone substitute manufacture.

#### 3.1.3. Continuous Digital Light Processing (cDLP)

**Figure 5:**
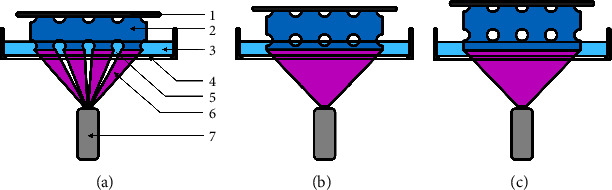
Schematic manufacturing process with a cDLP printer. (a–c) Continuous movement upwards during consistent projection. 1: print platform (constant movement along the z-axis); 2: scaffold; 3: resin; 4: glass plate; 5: dead zone; 6: projection; 7: projector (*x-*/*y*-axis).

The cDLP system is an adaptation of DLP printers and is based on the same working principle. However, instead of printing layer-by-layer, this technique produces a continuous layer-free scaffold. The build plate moves persistently upwards, leading to constant exposure of further space for polymerization (Figure 5). At the same time, the projection system emits a seamless stream of cross-sections of the print object, adjusted to the movement of the build platform [37]. In this case, it is necessary to have sufficient resin between the print subject and the window. The upwards movement of the construct generates a negative pressure that sucks in the surrounding resin. This prohibits the use of resins with viscosities higher than 10 Pa*∗* s. cDLP requires uncured resin between the object and window to prevent complete polymerization between the print object and the window (print failure). This is facilitated by letting oxygen pass through the window into the resin. The oxygen inactivates the crosslinking, leaving a thin layer of uncured resin called the “dead zone” behind [38].

A benefit, especially for bone substitute manufacturing, is the removal of the layer-to-layer interfaces. These interfaces are prone to shear fracturing. By eliminating them as a mechanical weakness, the structural integrity throughout the entire scaffold increases. However, due to the viscosity limit, the polymer density is restricted, thus capping the mechanical strength of each material indirectly. A direct comparison of the mechanical durability of bone scaffolds between DLP and cDLP has not yet been conducted.

#### 3.1.4. Two-Photon Polymerization (TPP)

**Figure 6:**
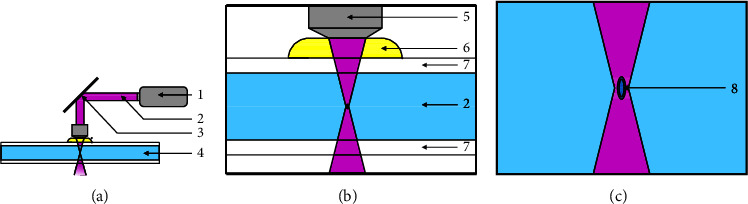
Schematic manufacturing process with a TPP printer. (a) Main components necessary for process; (b, c) close view during polymerization process; 1: femtosecond laser; 2: laser beam; 3: mirror; 4: photoresist / resin; 5: lens; 6: immersion oil; 7: glass plate; 8: voxel.

In contrast to previously described VP methods, the photosensitive material used in TPP is called a photoresist. These come in two variations: the negative photosensitive resist can start as a liquid that is polymerized into a solid, while the solid positive photoresist is turned into a liquid by the TPP process, creating a mold for casting. During the TPP process, a femtosecond laser beam is generated and focused into a small volume within the negative photoresist by the optical lenses and mirrors in which two or more photons are absorbed simultaneously by the photoinitiator, surpassing its specific activation energy threshold and subsequently solidifying the local polymers as ellipsoid-shaped building blocks, called voxels (Figure 6) [39]. The accuracy of the individual components makes TPP the VP method with the highest accuracy, allowing for a minimum feature size in the range of 100 nm.

The desired structure can be created by moving the focal point and polymerizing further photoresists. Unlike other printing techniques, this one is not layer-bound, which allows the manufacture of arbitrary isotropic 3D objects. However, in some cases, the polymerization of the photoresist has been shown to interfere with the passing of the laser through already printed parts, decreasing the accuracy and causing slight print deformation [40].

In TPP, a smooth surface is necessary to circumvent errors in the light's pathing. The printing vat is located between two glass plates (Figure 6). To reduce the differences in the refractory index, the distance between the optical system and the glass plate is bridged with immersion oil.

Multiple studies have described TPP as a highly precise polymerization method for the manufacturing of bone scaffolds, even in creating subcellular-sized details. In a study by Kampleitner et al., a scaffold manufactured from methacrylated poly (D,L-lactide-co-*ε*-caprolactone) showed limited *in vivo* bone formation after being implanted into a mouse [16]. A major downside of the TPP process for the treatment of critically sized bone defects is the height limitation. To adequately treat these defects, a scaffold has to span 1-2 cm [1]. However, TPP is commonly limited to the *µ*m to mm scale [41–43]. Currently, under investigation is a solution that involves adjusting the focal length, but it has not yet been applied to bone scaffold development [44]. Further research and adjustment to the printing technique are necessary to scale the size of the scaffold to clinically relevant sizes.

### 3.2. Ceramic Stereolithography (CSL)

Fundamentally, CSL is an adjustment of the resin composition by adding ceramic particles and can be utilized with the previously mentioned VP technologies. The ceramic particles are added to conventional resins and mixed into a suspension with the goal of increasing their mechanical properties [45]. Polymerization of such a ceramic suspension resin results in the so-called green body. The green body itself is analyzed as a bone substitute candidate as it allows for a cell-laden resin. Another possibility is the subsequent sintering of the green body, starting with the debinding step, whereby the cured polymers are removed by heat. During this step, shrinkage occurs, decreasing the size of the green body in all dimensions. A further heat increase starts the sintering process, binding the ceramic particles together. The sintering process of a 3D-printed green body increases the mechanical stiffness significantly, even surpassing the mechanical properties of traditionally sintered parts of the same composition [46]. To ensure printability and accuracy, several aspects must be taken into account when selecting the ceramic particles: the particles have to be smaller than the layer height to enable high print quality, and the ceramic granulates require low absorption of the wavelength used for the resin curing. Furthermore, the ceramic particles increase the viscosity of the resin, thus self-limiting the maximum concentration due to the viscosity restrains of SLA printing methods. In addition, the ceramic particles negatively impact the resolution by reflecting the curing light from the particles, resulting in overcuring, i.e., curing light outside the intended area. This problem can be addressed by the addition of dye to the resin that hinders the penetration depth of the curing light and decreases overcuring by limiting the consequences of light refraction [47, 48]. Simultaneously, the particles can lead to undercuring by partially absorbing the curing light. This insufficiently cured resin is subsequently removed together with the surrounding liquid/nonpolymerized resin. Thus, the composition and structure of the final scaffold are altered. These effects are reviewed in detail by Zakeri et al., giving further insights into CSL and the implications of ceramic particles in light-based manufacturing techniques [49].

However, the crucial aspect of mechanical resilience in bone substitute constructs benefits from the inclusion of ceramic granulate by increasing mechanical stability and enhancing osseous matrix formation in the bone scaffolds, even without sintering [50, 51]. If the bone scaffolds were produced with subsequent sintering, they showed, in some cases, a higher compressive strength. However, the shrinkage occurring during this step needs careful adjustment for the specific defect and prohibits the addition of cells within the resin. These cells can be added to the scaffold by seeding upon the surface, leading to the production of extracellular matrix and mineral deposition [52, 53]. Zhang et al. observed that the addition of micropores on the scaffolds surface led to increased bone density. The micropore's size and amount could be altered by varying the ceramic concentration within the resin as well as the sintering temperature [54].

### 3.3. Multimaterial Vat Photopolymerization

**Figure 7:**
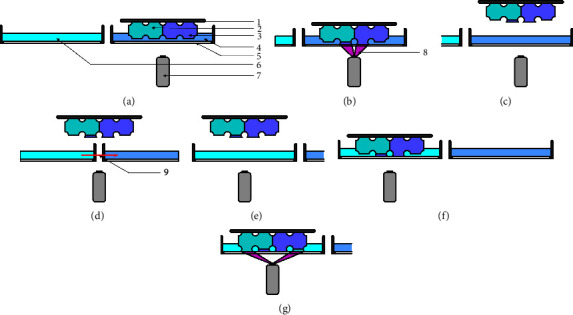
Multimaterial VP based on a DLP system. (a) End of a previous layer after movement of the print platform for the next layer; (b) projection of new layer; (c–e) raising of the print platform and movement of the second vat underneath the construct; (f) lowering into the second vat; (g) polymerization of next layer. 1: print platform; 2: scaffold material A; 3: scaffold material B; 4: resin B; 5: glass plate; 6: resin A 7: projector; 8: projection; 9: movement vector.

Modifying SLA and DLP printers through the addition of supplementary vats filled with different resins enables the successive printing of multiple materials or compositions within a single construct. After curing a layer in a resin bath (Figures 7(a) and 7(b)), the whole printing platform and the attached scaffold are moved into another vat filled with a different resin (Figures 7(c)–7(f)). Therefore, the photopolymerization adds a second material to the construct (Figure 7(g)). By combining these methods, it is feasible to fabricate hybrid scaffolds that enable a multitude of complex structures [24]. However, there are few studies in which these techniques have been applied to the development of bone scaffolds, mainly due to the lack of commercially available polymerization printers with multiple vats and materials.

**Figure 8:**
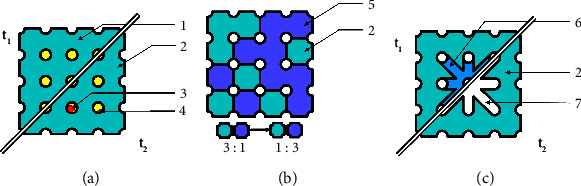
Hybrid bone scaffold compositions. (a) *t*_1_: initial scaffold containing a mechanically stable scaffold with a cell-supporting matrix within the pores, *t*_2_: later time point after implantation when vascular and cellular ingrowth into the supporting matrix occurred; 1: cell-friendly hydrogel; 2: scaffold; 3: cells; 4: blood vessel. (b) Combination of materials with different elasticity and compressive strength; 5: additional material with varying mechanical properties. (c) Two materials with different biodegradation speeds: *t*_1_: initial state with both materials present and *t*_2_: later time point after implantation when one material degraded; 6: fast dissolving material; 7: recovered space for tissue development.

Especially, bone scaffolds can benefit from utilizing different materials. The requirements for the scaffold materials could be separated into either providing mechanical stability or a cell-friendly environment. The polymer density and material selection required for mechanical stability prohibit cellular growth within the material before degradation. On the other hand, there are polymerizable hydrogels that provide osteoblasts and endothelial cells with an extracellular matrix that allows for proliferation and migration into the material but does not meet the physical requirements for load-bearing applications. A feasible solution to this challenge could be the combination of both categories of materials into a multimaterial hybrid scaffold. This first proposed hybrid scaffold, illustrated in Figure 8(a), assigns the task of cell interaction and physical resistance to different parts of the scaffold. The concept of hybrid scaffolds could additionally allow for other task-specific combinations of materials.

The combination of multiple vats to allow materials with different mechanical properties within one scaffold is depicted in Figure 8(b); Borrello et al. combined two distinctive formulations of ethylene glycol phenyl ether acrylate (EGPEA) with elastic moduli of 0.6 MPa and 33 MPa into a checkered arrangement [55]. Thus, the resulting scaffolds can be adjusted to the mechanical properties of the defect site by introducing mechanical gradients corresponding to the expected physiological strain. Critical to this combination of materials is the seamless connection between the materials, which enables load transfer without damaging the construct [55–57].

The combined materials can also differ in their individual degradation rates, as illustrated in Figure 8(c). In a multimaterial print study, the varying degradation speeds were depicted by the different release times of the six encapsulated drugs [58]. In the field of bone substitute manufacturing, the staggered degradation of the scaffold negates the risk of premature absorption of the scaffold. The use of materials with faster degradation rates increases bone formation, as shown by Nettleton et al. [59]. However, choosing materials with shorter degradation times increases the risk of complete degradation before sufficient replacement with new bone. The combination of a slow-absorbing material that provides mechanical support and a fast-degrading material that accelerates bone formation could bring together the best of both worlds. The ingrowth of bone into the scaffold could also strengthen the connection between the scaffold and the bone tissue by embedding one into the other.

This approach also enables printing with sacrificial materials. This allows structural support of fragile structures, such as the lumen in vascular architecture, during the printing process. Thomas et al. used enzymatically degradable sacrificial materials to create perfusable channels lined with endothelial cells [60].

Hybrid scaffolds could take advantage of the high printing accuracy granted by VP techniques to add one or more aspects to the scaffold. The manufacturing process can also include other additive manufacturing techniques, like FFF, to produce multimaterial scaffolds [61].

### 3.4. Materials for Vat Photopolymerization of Bone Substitutes

The resins used in VP processes consist of several materials and additives. Monomers and oligomers with reactive handles are the foundation of the resin. Exposure to light of the visible and ultraviolet spectrum leads to the formation of covalent bonds, resulting in polymers. These polymers can possess very different properties: on the one hand, soft and cell-friendly support is given by compositions like methacrylated gelatin (GelMA) [62, 63], collagen, and chitosan [43, 64]. On the other hand, mechanically stiff materials like polycaprolactone (PCL) [65] or polylactic acid (PLA) [66, 67] result in mechanically stiff structures that do not support cellular growth. To facilitate the crosslinking process, so-called photoinitiators are added to the resin. Photoinitiators absorb photons until they reach their excited state and decompose into radicals. These react with the monomers to form monomer radicals, which subsequently fuse with another monomer radical [31, 68]. Commonly used biocompatible photoinitiators include lithium phenyl-2,4,6 trimethyl-benzoyl phosphinate (LAP) [69], Irgacure® variants [59], or Lucirin® TPO-L [47].

Due to the viscosity limitations (Table 2) of the various vat polymerization techniques, a method to alter the resin's viscosity is required. Lowering the viscosity can be achieved by heating the resin during the print process or by adding solvents. These solvents are categorized as either reactive or nonreactive diluents. The key difference is that the reactive diluents react during polymerization and undergo crosslinking [70]. High concentrations of reactive diluents can affect the crosslinking density within the construct, leading to inhomogeneous crosslinking networks and decreased mechanical properties [71, 72]. Furthermore, vinyl-based solvents like N-vinylpyrrolidone can react with nonbiodegradable polymers, limiting their applicability [73]. Diethyl fumarate, on the contrary, is a member of the reactive diluents, which form biodegradable compounds [30]. Nonreactive diluents stay chemically inert and do not interact with the crosslinking network. Thus, the diluent can be removed after the printing process. Dienel et al. extracted the diluent propylene carbonate by immersing the construct in a propylene carbonate-ethanol solution and subsequently increasing the ethanol concentration stepwise. This controlled removal process slows the shrinkage process of the scaffold [47].

A further possible additive for a resin is a dye used to adjust the curing depth during printing by absorbing the curing light [74]. Depending on the photoinitiator and wavelength for photopolymerization, differing dyes are applied. For absorbing UV rays, a dye of the hydroxyphenyl benzotriazole class can be utilized, while thermoplastic yellow 104 (BASF, USA) absorbs light in the visible wavelength range. By increasing the dye concentration, the cure depth and light sensitivity of the resin can be adjusted.

In CSL, the resin is further modified through the implementation of ceramic powder into the resin. Ceramics such as SiO_2_ [75], hydroxyapatite (HA) [76, 77], and other calcium-phosphate particles are used to develop bone substitutes.

## 4. Applicability

Throughout the publications, a wide variety of biologically compatible materials were used to manufacture the constructs. However, their applicability *in vivo* was only assessed in 13 of the 41 analyzed publications. A detailed depiction of the biological aspects of the individual studies and their impact on final results is listed in Appendix 1. A key metric analyzed to determine the construct's applicability was the mineralization *in vitro* and the increased bone formation compared to their controls *in vivo* in 12 of the 13 studies.

The structural aspects of the reviewed bone scaffolds, such as their mechanical properties, and architectural factors like scale, porosity, pore size, and print accuracy, are crucial for bone substitute manufacturing. The compressive strength before failure of constructs with ceramic particles ranged from 0.36 MPa [78] up to 44 MPa [79], while the highest observed compressive strength of ceramic-free constructs was <1.2 MPa [16, 80]. In papers regarding constructs with an addition of solid particles to the resin, a deviation of the final scaffold from the planned design was observed. This deviation was particularly visible in the change in porosity, which increased by up to 5% in some studies and decreased by up to 23% in others [47, 78]. These variations were attributed to different mechanisms by the authors, such as solvent removal, light scattering, and shrinkage due to the heat during the sintering process (Table 3).

**Figure 9:**
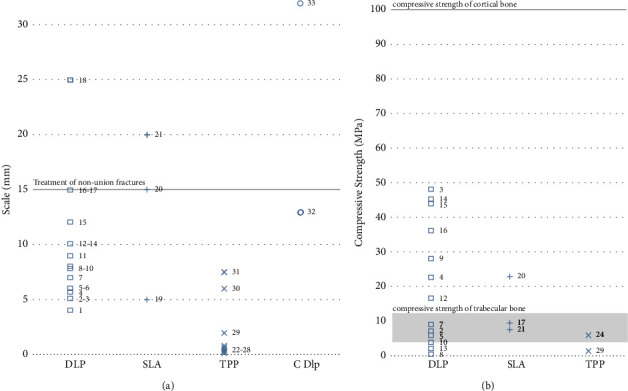
(a) Scale of the constructs compared to the defined lower limit of a nonunion fracture; (b) a representation of the compressive strength (in MPa) with a reference line at the physiological compressive strength of bone. References: 1: [69]; 2: [54]; 3: [91]; 4: [93]; 5: [85]; 6: [19]; 7: [53]; 8: [78]; 9: [75]; 10: [87]; 11: [86]; 12: [89]; 13: [90]; 14: [92]; 15: [79]; 16: [88]; 17: [47]; 18: [18]; 19: [82]; 20: [50]; 21: [98]; 22: [97]; 23: [43]; 24: [42]; 25: [96]; 26: [40]; 27: [94]; 28: [95]; 29: [16]; 30: [66]; 31: [41]; 32: [59]; and 33: [30].

Two crucial considerations in bone graft substitute manufacturing are depicted in Figure 9. Quantitative data from the publications discussed in this review are plotted in Figure 9(a) for size and Figure 9(b) for mechanical strength. The different printing technologies are listed on the *x*-axis. In each case, the required parameters are also defined on the *y*-axis. In (a), the size of a critically sized fracture is marked at 15 mm, whereas in (b), the compression load capacity of cortical and trabecular bone is shown as a reference bar. The parameter used in this review to assess the scale of the construct is the longest measurement along an axis within a construct. If this value is compared between the publications, a difference is noticeable depending on the printing technique used. There is a noteworthy difference between the SLA, DLP, and cDLP processes with a range of 4 mm to 32 mm (average: 11.41 mm; standard deviation: 7.22 mm) and the constructs manufactured by TPP with a span of 90 *µ*m to 7.5 mm (average: 1.93 mm; standard deviation: 2.65 mm) [30, 41, 62, 97]. This variance is seen in particular in the *z*-axis, where the TPP-produced constructs showed a value between 30 *µ*m and 230 *µ*m (average: 103 *µ*m; standard deviation: 73.8 *µ*m). In addition to size limitation, TPP also differs from the other three printing methods in other respects. For instance, the inherently unique approach facilitates a nonlayer-by-layer approach. In combination with the increased accuracy, the TPP offers a high degree of freedom in the scaffold design. Skoog et al. exploited this TPP-specific capability to create additional microarchitecture on the surface of the constructs. As a result, they were able to determine the alignment of the cells on the interface [99]. However, TPP is limited by the requirements of the photoinitiator and the materials. In contrast, DLP and SLA printing methods allow a much wider range of materials, including ceramic particles. DLP printing systems are used particularly frequently for CSL, in 13 out of 17 cases. These differ from SLA printers mainly by having a shorter printing time at the expense of a minimally reduced print resolution. The accuracy of the printers, especially in terms of porosity and pore size, is relevant for the production of bone substitutes. However, all VP technologies can produce porosities from 50% to a solid framework with pore sizes from 10 *µ*m to 1.4 mm. Therefore, when selecting between available VP technologies, this accuracy should only be of concern for specific applications.

## 5. Translational Considerations and Future Directions

In this paper, we discussed the current VP methods and publications concerning the required properties for the clinical application of bone substitute products with an emphasis placed on biocompatibility, printability, the effects of the scaffold's architecture, and the numerous variables of the scaffold's mechanical load-bearing capacity.

Fundamental prerequisites for the used materials are biocompatibility and nonimmunogenicity *in vivo*. A whole range of materials utilized in VP showed little to no negative effects on the constructs' surrounding area. Despite the utilization of photoinitiators and the corresponding UV/Visible light, the cells present in the resin showed only limited effects on their vitality rate (Appendix 1). However, several constructs have not yet been studied *in vivo* and thus do not allow for a statement on their immunogenicity, requiring additional research [40, 65, 84].

To facilitate fast remobilization of the patient, the bone substitute must possess mechanical properties surpassing or equaling the physiological requirements at the defect site. In adult patients, the mechanical strength needed is in the range of 100 to 200 MPa [21]. In the case of biodegradable implants, limited mechanical strength for daily activities could only be tolerated temporarily until the scaffold has been replaced and the mechanical load-bearing capacity recovers. However, to prevent failure of the construct, physiological load-bearing capacity is preferable. Despite various improvements, none of the reviewed papers observed mechanical resistance similar to human cortical bone (Figure 9). A fundamental problem in the development of bone constructs is the balance between a load-bearing matrix and the maintenance of a cell-supportive environment. The stability of the scaffold is determined by the compressive and elastic forces before its failure and is a key metric to determine the applicability of a scaffold. In this context, the scaffold's fatigue resistance under repeated loads is particularly important for clinical application. However, this resilience is the most difficult part to achieve with VP manufacturing techniques due to the correlation between polymer density/mechanical strength and the technique's limited viscosity range (Table 2). In the reviewed literature, various techniques were applied to increase the load-bearing capabilities of the scaffolds. The main determinants impacting the mechanical strength were alterations to the scaffolds architecture and the materials used (Table 3).

A key characteristic of the construct's biodegradation capability directly affects the scope of the scaffold's clinical application. For example, it would be of particular interest in adolescents and younger patients to allow reabsorption of the bone substitute and subsequent replacement with the patient's bone tissue. However, the risk of mechanical failure due to a mismatch of biodegradation and bone matrix synthesis is inherent to these materials. Thus, there is clinical and scientific relevance for both biodegradable and bioinert materials. On the one hand, there are hydrogels such as GelMA, collagen, and chitosan, which have a half-life of only weeks to months, enabling rapid cellular ingrowth and relatively long diffusion distances for nutrients and waste exchange [100]. More rigid biodegradable materials, such as tPLA and PLCL, showed increased mechanical resilience of >1.2 MPa at 20% strain [80]. In an experiment by Nettleton et al., a correlation between degradation rate and bone formation rate was observed. The shorter the degradation period is, the sooner the bone formation occurs [59]. Nevertheless, these materials do not meet physiological mechanical requirements. On the other hand, combining acrylester polymers with ceramic particles showed significantly higher mechanical resistance, best approximating the required properties, with ultimate failure occurring above 5 MPa [46].

However, in the case of multiple studies, the research focus was on the biological assessment of the printed constructs instead of their mechanical resistance, leading to a gap in the data for many of the reviewed papers. A further difficulty is that throughout the publications, the data on mechanical resilience showed immense differences depending on the analysis methods. In addition, the analysis was mainly one-directional, e.g., a clamping device, and lacked data on torsion resistance or nonaxial strain. In several papers, the mechanical characterization evaluated only the failure load of the construct and not the strength under repeated load cycles. In some cases, only simulations of the theoretical mechanical resilience were provided. To determine the applicability of the bone constructs for clinical use, this crucial information is needed to assess the chance of constructing failure after surgical implementation.

A further impact on the mechanical as well as biological properties of the bone substitute constructs is the architecture, enabled by the high resolution of VP techniques and the sufficient printability of the materials. Some aspects must be facilitated by the manufacturing process to permit the production of a suitable architecture so that the construct can be optimally integrated into the existing bone matrix. Multiple studies emphasized the benefit of an interconnected porosity to increase osteogenic differentiation and mineralization within the scaffold. In addition, this grants space to allow for vascularization and guided cell migration, leading to the integration and ingrowth of bone into the construct. With their high spatial resolution, all VP methods can sufficiently fulfill these design requirements. Porosities in the range of 50% up to >90% were obtained with pore sizes ranging from 20 *µ*m to >1 mm according to the specifications of the study authors (Table 3). Thus, VP allows a high degree of internal architectural freedom in the development of bone constructs. However, external size dimensions have to be met for clinical use. Since the body heals bone defects of less than 1.5 cm even without the insertion of material, this size requirement must be surpassed by the scaffolds. The application of bone scaffolds focuses on the treatment of larger defects and thus requires a minimal size of the bone constructs. This restriction applies, in particular, to highly accurate TPP because, despite research to increase the maximum size of the scaffolds, no clinically relevant studies have yet been published with this technology [44]. Although SLA, DLP, and cDLP are capable of fabricating scaffolds larger than 1.5 cm, few publications have applied this capability to fabricate scaffolds larger than 1.5 cm (Figure 9 and Table 3).

To validate the accuracy of the printing process and to determine the effects of environmental influences, the deviation of the printed construct from the CAD model can be analyzed [101]. Either a negligible or a predictable deviation is crucial in the case of bone substitute manufacturing since the implant has to fit the patient's bone defect precisely to negate incorrect loading of the adjacent tissues and incorrect posture with long-term implications. Thus, even minor inaccuracies could negatively affect patient outcomes. However, the defining feature of VP manufacturing techniques compared to other AM methods is superior resolution; hence, irregular deformation can negate this advantage.

These deviations varied depending on the printing method and material, as listed in Table 3, and are especially pronounced in constructs fabricated with ceramics due to shrinkage during the debinding and sintering process accompanied by a decrease in porosity that affected the constructs in several papers [53, 78, 79]. However, these manufacturing-dependent divergences are known and taken into consideration during the design and fabrication processes. At the same time, a deviation in the opposite direction may occur due to the addition of ceramic particles to the resin. Since the insertion of ceramic granulate increases the resin's viscosity, a diluent is added in some cases to facilitate the manufacture despite the viscosity limitations of the printing technique. In the case of Dienel et al., this led to a 5% increase in the final porosity of the scaffold after the removal of the diluent after the printing process [47]. If, however, the construct cannot withstand the alterations to its structure during the evaporation of liquid, a slight deformation occurs [94].

Since no single resin can fulfill all criteria simultaneously, a compromise of these 3 basic prerequisites of printability, biocompatibility, and mechanical properties is necessary to produce an optimal bone construct with VP technologies. The technology of multimaterial VP could address several problems. As demonstrated by other multimaterial printing methods such as multihead extrusion, the materials could consequently offer complementary advantages [102]. By developing a multimaterial VP technique, the specific benefits of photopolymerization could be utilized in a similar fashion. However, only a few studies with multimaterial VP have been published to date, leaving limited conclusions with only anecdotal data.

## 6. Conclusion

Over the past years, multiple papers utilizing VP manufacturing for bone defect treatment have been published. A wide range of materials has been assessed for bone construct manufacturing, many of which have been shown to be suitable for acting as an osteogenic environment. However, mechanical resilience currently does not equal or surpass the physiological load-bearing or tensile characteristics of bone. The high resolution of VP techniques enables complex internal architectures to optimize osteoconduction and defect-specific adaptation. Incorporating biodegradable materials into the design enables adjustable resorption of the scaffold and allows the ingrowth and replacement by the patient's bone, consequently relieving the scaffold of its mechanical tasks. To further bridge the gap between mechanical and biological requirements, a hybrid scaffold can combine multiple materials into a single, cell-accommodating, mechanically resistant, and biodegradable construct.

## Figures and Tables

**Table 1:** Inclusion/exclusion criteria.

Inclusion criteria	Exclusion criteria
(i) Focus on bone scaffold	(i) Before 2010/after September 2022
(ii) *In vitro/in vivo* studies	(ii) Unavailability in English
(iii) Manufacture with VP techniques	(iii) No biological assessment

**Table 2:** Overview of the various VP methods.

	SLA	DLP	cDLP	TPP
Size scale	mm-cm	mm-cm	mm-cm	*µ*m-mm
Minimal feature size	85 *µ*m	35 *µ*m–100 *µ*m	50 *µ*m	100 nm
Print deviation	<50 *µ*m	>100 *µ*m	>100 *µ*m	<1 *µ*m
Layer height	25 *µ*m–100 *µ*m	50 *µ*m	Continuous	Not layer bound (voxel)
Material viscosity	<5000 MPa*∗* s	100–8000 MPa*∗* s	<10 Pa*∗* s	Independent
Applicability for bone substitute printing	Yes	Yes	Yes	Limited (*z*-axis restriction)
References	[25, 26]	[26–28]	[29, 30]	[31]

Most values represent optimal conditions. SLA: stereolithography; DLP: digital light processing; cDLP: continuous digital light processing; TPP: two-photon polymerization.

**Table 3:** Depiction of the materials used in VP processes.

Printing method	Application	Scale	Porosity	Pore size	Mechanical properties	Material	Sintering	Deviation from CAD to scaffold	Reason for deviation	Reference
SLA	*In vitro*	—	53%–78%	—	0.8 MPa–2.6 MPa^†^	PEGDA/HA	n	—	—	[81]
	*In vitro*	20 mm	51%–76%	160 *µ*m–380 *µ*m	8.2 MPa^‡^	HA/TCP/propenoic acid	y	—	—	[46]
	*In vitro*	5 mm	71%	500 *µ*m	5 MPa–9 MPa^‡^	HA/oligolactide	n	—	—	[82]
	*In vitro*	—	75%–90%	350 *µ*m	0.4 MPa–63 MPa^†^	EHA/IBOA	n	—	—	[83]
	*In vitro*	—	50%–80%	—	400 MPa–500 MPa^§^	Soybean oil epoxidized acrylate	n	—	—	[84]
	*In vitro*	15 mm	50%–80%	500 mm–1.2 mm	23 MPa^‡^	PEGDA/nHA	n	—	—	[50]

DLP	*In vitro*	15 mm	83%	1.3 mm	—	PTMC/TCP	n	Average of 5% higher porosity	Removal process of the diluent	[47]
	*In vitro/in vivo*	7 mm	65%–70%	620 *µ*m	3.6–9 MPa^‡^	CPP	y	29% size decrease	Heat shrinkage	[53]
	*In vitro/in vivo*	6 mm	—	0.8 mm–1.4 mm	2.8 MPa–6.2 MPa^‡^	HA/TCP	y	—	—	[52, 85]
	*In vitro*	8 mm	55%	600 *µ*m	>5 MPa–28 MPa^‡^	CaSiO_3_-Mg/Sr; HDDA, PPTTA	y	5% variation in porosity	Heat shrinkage	[75]
	*In vitro*	12 mm	50%–75%	400 *µ*m	6–44 MPa^‡^	TCP	y	16% porosity reduction	Light scattering	[79]
	*In vitro*	7, 8 mm	77%	500 *µ*m–700 *µ*m	0.36 MPa^‡^	HA	y	23% porosity reduction	Shrinkage due to sintering	[78]
	*In vitro*	4 mm/0.6 mm^z^	Solid	Solid	—	GelMA	n	—	—	[62]
	*In vivo*	9 mm	70%	720 *µ*m	—	PTMC/HA	n	27% (open pores); 14% (closed pores)	Support structures resulting from personalized adaptation	[86]
	*In vitro/in vivo*	5 mm	—	200 *µ*m–300 *µ*m	5–7 MPa	TCP	y	—	—	[54]
	*In vitro/in vivo*	—	67%	600 *µ*m	3.6 MPa	BCP	y	17–21% size decrease	Shrinkage due to sintering	[87]
	*In vitro*	15 mm/8 mm	55%–60%	—	25 MPa–36 MPa	SWCNT/HA/“bioresin”	n	—	—	[88]
	*In vitro/in vivo*	5 mm/10 mm	56%–64%	400 *µ*m–600 *µ*m	10.4–16.5 MPa	TCP	y	≤5% (*X*-*Y* direction); ≤8.5% (vertical axis)	Anisotropic shrinkage due to sintering	[89]
	*In vivo*	25 mm	74%–76%	800 *µ*m	—	PTMC + TCP	n	—	Expected shrinkage during solvent extraction	[18]
	*In vitro*	10 mm/5 mm	60%	400 *µ*m	2 ± 1.2 MPa	PEGDA/Si-CaP	n	—	—	[90]
	*In vitro/in vivo*	5 mm/3 mm	—	400 *µ*m	3.5–8 MPa (greenbody); 19–48 MPa (sintered)	HA	y	0,1 mm (greenbody manufacture)/5%–25% (sintered)	Shrinkage due to sintering	[91]
	*In vitro/in vivo*	10 mm/4 mm	58%	480–700 *µ*m	35–45 MPa	CSi-Mg6	y	9%–15% decrease in porosity	Shrinkage due to sintering	[92]
	*In vitro*	5.5 mm/1.8 mm	70%	670 *µ*m–1180 *µ*m	3–22.5 MPa	nHA	y	25%–30% decrease in porosity	Shrinkage due to sintering	[93]
	*In vitro/in vivo*	6 mm	70%	700 *µ*m–800 *µ*m	—	PTMC/HA	n	Deviation increases with the addition of HA	—	[19]

cDLP	*In vitro/in vivo*	13 mm	82%	621 *µ*m	—	PPF	n	—	—	[59]
	*In vitro*	32 mm/1.2 mm^z^	Solid	Solid	180 Mpa^†^	PPF	n	≤0,8% increase in size	Deviations are consistent and can be adjusted	[30]

TPP	*In vitro*	32 mm/1.2 mm^z^	Solid	Solid	180 Mpa^†^	PPF	n	≤0,8% increase in size	Deviations are consistent and can be adjusted	[30]
	*In vitro/in vivo*	2 mm	—	300 *µ*m	<1.2 Mpa^‡,1^	PLCL	n	8% pore size deviation	—	[16, 80]
	*In vitro*	7,5 mm/230 *µ*m^z^	—	40 *µ*m–180 *µ*m	6 MPa–119 Mpa^¶^	LC; UDMA	n	—	—	[41]
	*In vitro*	600 *µ*m/40 *µ*m^z^	86%–92%	160 *µ*m	—	IP-L780	n	Some microtubes transitioned from circular to quasi hexagonal	Superficial tension during developing and drying	[94]
	*In vitro*	200 *µ*m/50 *µ*m^z^	—	25 *µ*m	3 Mpa–6 Mpa^‡^	IP-Dip/TiO_2_ coat	n	—	—	[42]
	*In vitro*	750 *µ*m/80 *µ*m^z^	—	—	—	Poly (D,L-lactide)	n	—	—	[95]
	*In vitro/in vivo*	6 mm/100 *µ*m^z^	69%	—	—	tPLA	n	4%–8% higher porosity	Variations in wall thickness	[66]
	*In vitro*	480 *µ*m/170 *µ*m^z^	—	35 *µ*m	—	PETA/BisGMA	n	Slight deformation in the third layer	Laser passed through already printed parts	[40]
	*In vitro*	300 *µ*m^z^	54%–65%	150 *µ*m–250 *µ*m	—	ZPO/MAPTMS	n	—	—	[96]
	*In vitro*	90 *µ*m/30 *µ*m^z^	—	10 *µ*m–30 *µ*m	—	ZPO/MAPTMS	n	—	—	[97]

z: along *z*-axis; †: tensile modulus; ‡: compressive strength; §: compressive modulus; ¶: dynamic modulus; 1: at 20% strain; HA: hydroxyapatite; TCP: tricalcium phosphate; BCP: biphasic calcium-phosphate; PTMC: poly(trimethylene carbonate); ZA: zoledronic acid; PEGDA: polyethylene glycol diacrylate; EHA: 2-ethylhexyl acrylate; IBOA: isobornyl acrylate; GelMA: methacrylated gelatin PDA: poly(dopamine); PPF: poly(propylene fumarate); tECM: tendon extracellular matrix; HDDA: 1,6-hexanediol diacrylate; PPTTA: ethoxylated pentaerythritol tetraacrylate; Col: collagen; CT: chitosan; LC: D,L-lactide-co-*ε*-caprolactone copolymers; UDMA: urethane dimethacrylate; UDA: urethane diacrylate; IP-L780: product name by Nanoscribe; IP-Dip: product name by Nanoscribe; tPLA: tetrafunctional poly(D,L-lactides); PLCL: methacrylated poly(D,L-lactide-co-*ε*-caprolactone); PETA: pentaerythritol triacrylate; BisGMA: bisphenol A-glycidyl methacrylate; ZPO: zirconium isopropoxide; MAPTMS: methacryloxypropyl trimethoxysilane; SWCNT: single-walled carbon nanotubes.

## Data Availability

The data supporting this systematic review are from previously reported studies and datasets, which have been cited.

## Supplementary Materials

Supplementary Materials Appendix 1 lists a detailed depiction of the biological aspects of the individual studies and their impact on final results.
