# Supplementary material for: 3D Printing of Bone Substitutes Based on Vat Photopolymerization Processes: A Systematic Review
Source: J Tissue Eng Regen Med. 2023 Apr 8;2023:3901448. doi: 10.1155/2023/3901448 (PMC11918515; doi:10.1155/2023/3901448)
Supplement: Supplementary Materials — Appendix 1 lists a detailed depiction of the biological aspects of the individual studies and their impact on final results. [file 3901448.f1.docx]

**Appendix**

**3D printing of bone substitutes based on vat polymerization processes – a systematic review**

Simon Enbergs ^1,^*, Jacob Spinnen^1^, Tilo Dehne^1^ and Michael Sittinger^1^

^1^ Tissue Engineering Laboratory, BIH Center of Regenerative Therapies, Department of Rheumatology & Clinical Immunology, Charité - Universitätsmedizin Berlin, Charitéplatz 1, 10117 Berlin, Germany

* Correspondence: simon.enbergs@charite.de

**Appendix 1** Depiction of the materials used in VP processes for bone substitute manufacture and their properties.

| **Printing Method** | **Material** | **Observed Parameters** |  | **Conclusion** |  | **Ref** |
| --- | --- | --- | --- | --- | --- | --- |
| SLA | 60% HA / 40% TCP  100% HA | *in vitro (MG-63)*: biocompatibility, cell growth, and morphology; *in vivo (rat model)*: inflammation, osseointegration |  | *in vitro*: cell viability of >75%; *in vivo*: new bone formation occurred in the pores without signs of inflammation |  | (Le Guéhennec et al., 2020) |
|  | 60% PTMC / 40% HA  60% PTMC / 40% TCP | *in vitro:* cell viability and biocompatibility; *in vivo:* bone formation and mineralization, observing the influence of BMP and ZA on surrounding tissue |  | *in vivo:* bone formation of implanted groups was improved compared to an empty control group; addition of bioactive BMP and ZA lead to increased mineralization |  | (Teotia et al., 2020) |
|  | HA / TCP / Propenoic acid | *proof of concept:* compressive strength, printability |  | the compressive strength in the printed scaffold after debinding and sintering was higher compared to traditionally sintered biomaterials |  | (Mangano et al., 2019) |
|  | HA / oligolactide | *in vitro:* mechanical properties and gentamicin drug release |  | The scaffold had a compressive strength of over 5 MPa and the released drug followed a typical release profile of an initial burst followed by a slow release over the next few weeks. |  | (Channasanon, Udomkusonsri, Chantaweroad, Tesavibul, & Tanodekaew, 2017) |
|  | GelMA / nHA | *in vitro:* influence of co-cultured breast cancer cells on proliferation and osteoblast cytokine secretion and morphology |  | The proliferation of the osteoblasts in co-culture with breast cancer cells was inhibited and the alkaline phosphate expression decreased in comparison to the control group. |  | (Zhou, Zhu, et al., 2016) |
|  | PEGDA / HA | *in vitro:* biocompatibility, mineral deposition, the impact of low-intensity pulsed ultrasound stimulation |  | Both biocompatibility, as well as expression of osteogenic differentiation, could be observed. The ultrasound stimulation improved the mineral deposition. |  | (Zhou, Castro, et al., 2016) |
|  | EDA / IBOA | *in vitro:* biocompatibility, osteogenic differentiation compared between varying concentrations of EHA and IBOA |  | Osteogenic differentiation could be observed in all differing compositions; the mechanical properties, however, differed between the probes. |  | (Owen et al., 2016) |
|  | soybean oil epoxidized acrylate | *in vitro:* biocompatibility, cell attachment, and proliferation |  | The scaffolds were biocompatible, and the organic resin showed no significant difference from PLA or PCL controls. |  | (Miao et al., 2016) |
|  | PEGDA / nHA | *in vitro:* biocompatibility, osteochondral matrix development compared between scaffold with and without containing nHA |  | An increased amount of osteogenic matrix development could be shown by the addition of nHA into the scaffold. |  | (Castro, O'Brien, & Zhang, 2015) |
| DLP | calcium phosphate | *in vitro:* cell behavior and viability, mechanical properties; *in vivo:* bone formation, angiogenesis, and adverse effects |  | *in vitro:* sufficient cell viability, attachment, proliferation after 7 days; *in vivo*: New bone formation and blood vessels were observed throughout the scaffold’s pores. |  | (Wei et al., 2020) |
|  | PPF | Chemical and physical properties of various polymer concentrations |  | The scaffold’s architecture and the molecular mass of the polymer showed significant influence on the biodegradation speed. |  | (Walker et al., 2017) |
|  | HA | *in vitro:* mechanical properties and shrinkage after sintering; biocompatibility |  | the compressive strength of the manufactured scaffold was over 10 MPa; biocompatibility was tested over a timespan of 2 days |  | (Zeng et al., 2018) |
|  | HA / TCP | *in vitro:* biocompatibility, mechanical properties; *in vivo:* bone formation |  | *in vitro:* materials showed no cytotoxicity; *in vivo (rabbit):* after 8 weeks the new bone formation covered 8%-10% of the defect area; *in vivo (beagle dog):* after 8 weeks, 20% of the defect site were covered by new bone formation |  | (Kim et al., 2020; Lim et al., 2020) |
|  | 60% PTMC / 40% HA | *in vivo (sheep):* biocompatibility and immunologic response, bone formation, biological functionality of the scaffolds |  | *in vivo*: moderate inflammatory reactions with the presence of immune cells; significant bone formation occurred, and the material showed not only osteoconductive but also exhibited osteoinductive properties in some of the HA-enriched samples |  | (Guillaume et al., 2020) |
|  | 60% PTMC / 40% HA | *in vitro (hBMSC):* biocompatibility, matrix deposition, osteogenic differentiation; *in vivo (rabbit):* bone formation |  | *in vitro:* addition of HA increased cell attachment and osteogenic differentiation, however, showed decelerated proliferation; *in vivo:* increased bone formation and angiogenesis |  | (Guillaume et al., 2017) |
|  | TCP | *in vitro:* biocompatibility, osteogenic behavior, mechanical properties |  | *in vitro:* high biocompatibility could be observed; depending on the design and porosity (50-75%) the compressive strength ranged between 6 and 44 MPa |  | (Schmidleithner et al., 2019) |
|  | HA | *in vitro:* biocompatibility, cell proliferation, toxicity, and impact of ceramic to resin ratio and sintering heat on micropore size*; in vivo:* mineral deposition and bone formation |  | *in vitro:* sintering heat affected micropore size significantly more than the initial ceramic concentration, sufficient viability and proliferation could be observed; *in vivo:* after 1 month the entire scaffold was filled with bone tissue |  | (B. Zhang et al., 2022) |
|  | TCP | *in vitro:* effect of surface roughness on osteogenic activity, biocompatibility, viability and proliferation*; in vivo:* bone formation |  | *in vitro*: viability of 95% and increased osteo-related gene expression; *in vivo*: an increased surface roughness led to higher bone mineral density and bone volume |  | (H. Zhang, Zhang, Xiong, Dong, & Li, 2021) |
|  | BCP | *in vitro:* biocompatibility, viability, effect of polydopamine coating and BMP-2 release, mineralization; *in vivo:* bone formation |  | *in vitro:* sufficient viability; Mineralization was observed throughout with the highest deposition in the sample with polydopamine coating and BMP-2; *in vivo:* Induced cell aggregation and new lamellar bone formation. |  | (Yang et al., 2022) |
|  | TCP | *in vitro:* biocompatibility, osteogenic differentiation markers; *in vivo:* bone formation and scaffold degradation |  | *in vitro:* strong cell attachment, good cell viability; *in vivo:* in the femoral defect new bone formation could be observed, less so in the intramuscular sample |  | (Xu et al., 2022) |
|  | PTMC / TCP | *in vivo:* difference between PTMC with and without TCP by clinical, radiological, and histological analysis of bone formation in critically sized defects |  | *in vivo:* both PTMC as well as PTMC + TCP showed comparable osteoconductive activity; in the PTMC + TCP implant histological signs of osteoinductive and inflammatory properties could be observed; the authors note the importance of research in large animal models since the results of small animal models differed |  | (K. Dienel et al., 2022) |
|  | SWCNT / HA | *in vitro:* mechanical properties and biocompatibility |  | *in vitro:* the addition of 1 - 2 wt% SWCNT to the bioresin + HA resin led to an increase in compressive strength; the cell viability in groups with and without SWCNT was observed to be >90% after 7 days |  | (Akbari-Aghdam et al., 2021) |
|  | PEGDA / Si-CaP | *in vitro:* mechanical properties, permeability, degradation, and apatite mineralization |  | *in vitro:* sufficient viability, mechanical strength and permeability could be observed; mineral deposition and gene expression showed an increased osteogenic differentiation |  | (Chen et al., 2022) |
|  | CSi‐Mg6 | *in vitro:* impact of pore sizes on mechanical properties and mineral deposition; *in vivo:* bone formation |  | *in vitro:* sufficient viability and mechanical properties; *in vivo*: depending on the pore size the bone formation varied; the most bone ingrowth could be observed in the scaffold with 600µm pore size at a porosity of 58% |  | (Qin et al., 2022) |
|  | nHA | *in vitro:* mechanical properties and cellular response |  | *in vitro:* good biocompatibility and mechanical properties similar to cancellous bone; however, the architecture of the scaffold effected the mechanical properties immensely: body-centric cubic structure 3 MPa to cubic pore shaped structure 22.5 MPa |  | (Liang et al., 2022) |
|  | PEGDA / tECM | *in vitro:* biocompatibility, cell activity, physical testing, influence on cell behavior by tECM; *in vivo:* repair assay in 4 mm rat calvarial defect model |  | *in vitro:* tECM positively influenced bio viability, increased osteogenic differentiation and migration / *in vivo:* improved bone formation in tECM scaffolds compared to PEGDA or empty control |  | (Luo et al., 2020) |
|  | PTMC / TCP | *proof of concept:* tensile strength, printability, microscopic analysis |  | optimal toughness could be achieved at a 51 wt % TCP effective concentration (353 J/m2), until a concentration of 50% TCP high printing quality was possible; higher concentrations were complicated by the viscosity limitations of SLA printing; close to object surfaces the concentration of TCP increased (surface enrichment) |  | (K. E. G. Dienel, van Bochove, & Seppälä, 2020) |
|  | CaSiO3-Mg/Sr; HDDA, PPTTA | *in vitro:* structural evaluation, mechanical properties, degradation differences with varying strontium concentrations |  | the structures consistently shrunk after sintering around 15-20%; the mechanical properties varied depending on the pore shape with cylindrical being the most stable (>16 Mpa); the strontium concentration can be applied to adjust the mechanical properties |  | (Li et al., 2021) |
|  | PCL; PDA / HA coating | *in vitro*: cell adhesion of hMSCs, osteogenic differentiation, and angiogenic differentiation with varying concentrations of CDA / HA coatings |  | The number of osteogenic and angiogenic molecular markers differed, increasing in correlation with the CDA / HA coating. |  | (Cheng, Chen, Wang, & Shie, 2016) |
|  | HA | *in vitro:* mechanical properties and cellular response |  | The structure experienced significant shrinkage of around 30% during sintering; no signs of cytotoxicity could be observed. |  | (Tesavibul et al., 2015) |
|  | GelMA | *in vitro:* cell viability, metabolic activity, mineralization, and gene expression of human mesenchymal progenitor cells isolated from differing body tissues |  | The cells within the construct remained viable. Periosteum-derived cells showed the highest mineral deposition and expressed genes associated with later stages of osteogenic differentiation. |  | (Amler et al., 2021) |
| cDLP | PPF (1kDa / 1,9kDa) | *in vitro:* biodegradation; *in vivo:* bone formation, inflammation, influence of biodegradation time on healing speed |  | *in vivo:* bone formation could be observed, with no signs of inflammation visible; faster biodegradation was accompanied with faster bone formation |  | (Nettleton et al., 2019) |
|  | PPF (1,1kDa / 2kDa) | *in vitro*: printability and mechanical properties |  | The PPF allowed for a high printing quality and exhibits an elastic modulus similar to human trabecular bone |  | (Luo, Le Fer, Dean, & Becker, 2019) |
| TPP | PLCL | *in vitro:* biocompatibility, immunological response, degradation, mechanical properties; *in vivo:* bone formation, immunological response |  | *in vitro:* limited osteogenic differentiation and high biocompatibility were observed, *in vivo:* no or limited bone formation observed with low inflammation |  | (Felfel et al., 2016; Kampleitner et al., 2020) |
|  | IP-L780, Col / CT coat | *in vitro (MG-63):* morphological assessment of print and coating, wettability, mineral deposition, and osteogenesis |  | high printing quality could be achieved; the coating with collagen and chitosan impacted and deformed the structural backbone; the wettability could be improved by the coating; optimal mineral deposition was observed with 20% Col/ 80% CT coat |  | (Păun, Mustăciosu, Popescu, Călin, & Mihăilescu, 2020; Paun, Popescu, Calin, et al., 2018) |
|  | LC; UDMA | *in vitro:* assessment of proliferation and differentiation with varying pore sizes, dynamic mechanical analysis |  | Optimal proliferation occurred at pore sizes of 60 µm to 100 µm with continuous vimentin expression. |  | (Florian et al., 2019) |
|  | IP-L780 | *in vitro:* cell differentiation and mineralization |  | Depending on the scaffold architecture, the mineralization varied heavily; it was observed that a higher interconnectivity increased the mineralization |  | (Paun, Popescu, Mustaciosu, et al., 2018) |
|  | IP-Dip / TiO_2_ coat | *in vitro:* mechanical properties, cellular response and mineral deposition |  | The scaffold exhibit exceptional mechanical stiffness; mineral deposition could be observed throughout, with the highest concentration on the mechanically compliant scaffold (0,7 MPa) |  | (Maggi, Li, & Greer, 2017) |
|  | tPLA | *in vitro*: biocompatibility, scaffold colonization; *in vivo:* bone formation, biodegradation |  | *in vitro:* no notable cytotoxicity could be observed; *in vivo (Kuznetsova et al., 2017):* after 10 weeks, 70% of the scaffold was filled with mineralized tissue and vascular ingrowth was visible; *in vivo (Timashev et al., 2016):* incorporation of the mice’s own MSCs into the scaffold increased bone formation and scaffold degradation |  | (Kuznetsova et al., 2017; Timashev et al., 2016) |
|  | poly (D,L-lactide) | *in vitro*: development of a photoinitiator of a ciprofloxacin derivative; mechanical properties and cell viability |  | *in vitro:* the photoinitator enabled accurate polymerization at a moderate energy absorbance; sufficent viability of >70% in all samples. |  | (Bardakova et al., 2021) |
|  | PETA / BisGMA | *in vitro:* wettability, biocompatibility, osteogenic differentiation |  | After being cultured in an osteogenic culture medium, the mesenchymal progenitor cells’ mineralized ECM could be observed; the cells exerted mechanical forces on the scaffold, leading to a slight deformation |  | (Heitz et al., 2017) |
|  | ZPO / MAPTMS | *in vitro:* biocompatibility, mineralization, gene expression |  | The in the scaffold immobilized BMP-2 showed a sustained release and led to a higher mineralization compared to the BMP-free control. |  | (Chatzinikolaidou et al., 2017) |
|  | Ormocomp® | *in vitro:* biocompatibility, osteogenic gene expression |  | cells adhered to the scaffold increased HA deposition; some upregulated gene expression could be shown |  | (Marino et al., 2014) |
|  | ZPO / MAPTMS | *in vitro:* biocompatibility, osteogenic differentiation, mineral deposition |  | The osteogenic differentiation and mineral deposition varied depending on the scaffold’s architecture, favoring a pore size of 150 µm. |  | (Koroleva et al., 2015) |
|  | UDA | *in vitro:* biocompatibility, cell proliferation and protein production |  | The scaffold showed biocompatibility, and the proliferation and protein production outpaced the cell culture control. |  | (Petrochenko et al., 2015) |
|  | ZPO / MAPTMS | *in vitro:* printability, biocompatibility, cellular response |  | high and consistent printing accuracy; cellular growth spanning the pores with a microarchitecture dependent orientation |  | (Skoog et al., 2014) |
|  | ZPO / MAPTMS | *in vitro:* biocompatibility, osteogenic differentiation |  | The niches are biocompatible and increased the osteogenic differentiation compared to the control. |  | (Nava et al., 2017) |
|  |  |  |  |  |  |  |

*Abbreviations: HA: hydroxyapatite; TCP: tricalcium phosphate; BCP: biphasic calcium phosphate; PTMC: poly (trimethylene carbonate); BMP: bone morphogenic protein-2; ZA: zoledronic acid; MG-63: immortalized osteoblast cell linage; PEGDA: poly (ethylene glycol) diacrylate; EHA: 2-ethylhexyl acrylate; IBOA: isobornyl acrylate; GelMA: methacrylated gelatin PDA: poly dopamine; PPF: poly (propylene fumarate); tECM: tendon extra cellular matrix; HDDA: 1,6- hexanediol diacrylate; PPTTA: ethoxylated pentaerythritol tetra-acrylate; hBMSC: human bone-marrow derived mesenchymal stem cell; MSC: mesenchymal stem cell; Col: Collagen; CT: chitosan; LC: D,L-lactide-co-ε- caprolactone copolymers; UDMA: urethane-dimethacrylate; UDA: urethane diacrylate IP-L780: product name by Nanoscribe; IP-Dip: product name by Nanoscribe; tPLA: tetrafunctional poly(D,L-lactides); PLCL: methacrylated poly(D,L-lactide-co-ε-caprolactone); PETA: pentaerythritol triacrylate; BisGMA: bisphenol A-glycidyl methacrylate; ZPO: zirconium isopropoxide; MAPTMS: methacryloxypropyl trimethoxysilane; Ormocomp®: product name by micro resist technology;* SWCNT: single walled carbon nano tubes;

**References**

Akbari-Aghdam, H., Bagherifard, A., Motififard, M., Parvizi, J., Sheikhbahaei, E., Esmaeili, S., . . . Khandan, A. (2021). Development of Porous Photopolymer Resin-SWCNT Produced by Digital Light Processing Technology Using for Bone Femur Application. *Arch Bone Jt Surg, 9*(4), 445-452. doi:10.22038/abjs.2020.43409.2189

Amler, A. K., Dinkelborg, P. H., Schlauch, D., Spinnen, J., Stich, S., Lauster, R., . . . Dehne, T. (2021). Comparison of the Translational Potential of Human Mesenchymal Progenitor Cells from Different Bone Entities for Autologous 3D Bioprinted Bone Grafts. *Int J Mol Sci, 22*(2). doi:10.3390/ijms22020796

Bardakova, K. N., Faletrov, Y. V., Epifanov, E. O., Minaev, N. V., Kaplin, V. S., Piskun, Y. A., . . . Timashev, P. S. (2021). A Hydrophobic Derivative of Ciprofloxacin as a New Photoinitiator of Two-Photon Polymerization: Synthesis and Usage for the Formation of Biocompatible Polylactide-Based 3D Scaffolds. *Polymers, 13*(19). doi:10.3390/polym13193385

Castro, N. J., O'Brien, J., & Zhang, L. G. (2015). Integrating biologically inspired nanomaterials and table-top stereolithography for 3D printed biomimetic osteochondral scaffolds. *Nanoscale, 7*(33), 14010-14022. doi:10.1039/c5nr03425f

Channasanon, S., Udomkusonsri, P., Chantaweroad, S., Tesavibul, P., & Tanodekaew, S. (2017). Gentamicin Released from Porous Scaffolds Fabricated by Stereolithography. *J Healthc Eng, 2017*, 9547896. doi:10.1155/2017/9547896

Chatzinikolaidou, M., Pontikoglou, C., Terzaki, K., Kaliva, M., Kalyva, A., Papadaki, E., . . . Farsari, M. (2017). Recombinant human bone morphogenetic protein 2 (rhBMP-2) immobilized on laser-fabricated 3D scaffolds enhance osteogenesis. *Colloids Surf B Biointerfaces, 149*, 233-242. doi:10.1016/j.colsurfb.2016.10.027

Chen, D., Chen, G., Zhang, X., Chen, J., Li, J., Kang, K., . . . Wang, X. (2022). Fabrication and in vitro evaluation of 3D printed porous silicate substituted calcium phosphate scaffolds for bone tissue engineering. *Biotechnol Bioeng*. doi:10.1002/bit.28202

Cheng, Y. L., Chen, Y. W., Wang, K., & Shie, M. Y. (2016). Enhanced adhesion and differentiation of human mesenchymal stem cell inside apatite-mineralized/poly(dopamine)-coated poly(ε-caprolactone) scaffolds by stereolithography. *J Mater Chem B, 4*(38), 6307-6315. doi:10.1039/c6tb01377e

Dienel, K., Abu-Shahba, A., Kornilov, R., Björkstrand, R., van Bochove, B., Snäll, J., . . . Mannerström, B. (2022). Patient-Specific Bioimplants and Reconstruction Plates for Mandibular Defects: Production Workflow and In Vivo Large Animal Model Study. *Macromol Biosci, 22*(4), e2100398. doi:10.1002/mabi.202100398

Dienel, K. E. G., van Bochove, B., & Seppälä, J. V. (2020). Additive Manufacturing of Bioactive Poly(trimethylene carbonate)/β-Tricalcium Phosphate Composites for Bone Regeneration. *Biomacromolecules, 21*(2), 366-375. doi:10.1021/acs.biomac.9b01272

Felfel, R. M., Poocza, L., Gimeno-Fabra, M., Milde, T., Hildebrand, G., Ahmed, I., . . . Liefeith, K. (2016). In vitro degradation and mechanical properties of PLA-PCL copolymer unit cell scaffolds generated by two-photon polymerization. *Biomed Mater, 11*(1), 015011. doi:10.1088/1748-6041/11/1/015011

Florian, B., Michel, K., Steffi, G., Nicole, H., Frant, M., Klaus, L., & Henning, S. (2019). MSC differentiation on two-photon polymerized, stiffness and BMP2 modified biological copolymers. *Biomed Mater, 14*(3), 035001. doi:10.1088/1748-605X/ab0362

Guillaume, O., Geven, M. A., Sprecher, C. M., Stadelmann, V. A., Grijpma, D. W., Tang, T. T., . . . Eglin, D. (2017). Surface-enrichment with hydroxyapatite nanoparticles in stereolithography-fabricated composite polymer scaffolds promotes bone repair. *Acta Biomater, 54*, 386-398. doi:10.1016/j.actbio.2017.03.006

Guillaume, O., Geven, M. A., Varjas, V., Varga, P., Gehweiler, D., Stadelmann, V. A., . . . Eglin, D. (2020). Orbital floor repair using patient specific osteoinductive implant made by stereolithography. *Biomaterials, 233*, 119721. doi:10.1016/j.biomaterials.2019.119721

Heitz, J., Plamadeala, C., Wiesbauer, M., Freudenthaler, P., Wollhofen, R., Jacak, J., . . . Marksteiner, R. (2017). Bone-forming cells with pronounced spread into the third dimension in polymer scaffolds fabricated by two-photon polymerization. *J Biomed Mater Res A, 105*(3), 891-899. doi:10.1002/jbm.a.35959

Kampleitner, C., Changi, K., Felfel, R. M., Scotchford, C. A., Sottile, V., Kluger, R., . . . Epstein, M. M. (2020). Preclinical biological and physicochemical evaluation of two-photon engineered 3D biomimetic copolymer scaffolds for bone healing. *Biomater Sci, 8*(6), 1683-1694. doi:10.1039/c9bm01827a

Kim, J. W., Yang, B. E., Hong, S. J., Choi, H. G., Byeon, S. J., Lim, H. K., . . . Byun, S. H. (2020). Bone Regeneration Capability of 3D Printed Ceramic Scaffolds. *Int J Mol Sci, 21*(14). doi:10.3390/ijms21144837

Koroleva, A., Deiwick, A., Nguyen, A., Schlie-Wolter, S., Narayan, R., Timashev, P., . . . Chichkov, B. (2015). Osteogenic differentiation of human mesenchymal stem cells in 3-D Zr-Si organic-inorganic scaffolds produced by two-photon polymerization technique. *PLoS One, 10*(2), e0118164. doi:10.1371/journal.pone.0118164

Kuznetsova, D., Ageykin, A., Koroleva, A., Deiwick, A., Shpichka, A., Solovieva, A., . . . Timashev, P. (2017). Surface micromorphology of cross-linked tetrafunctional polylactide scaffolds inducing vessel growth and bone formation. *Biofabrication, 9*(2), 025009. doi:10.1088/1758-5090/aa6725

Le Guéhennec, L., Van Hede, D., Plougonven, E., Nolens, G., Verlée, B., De Pauw, M. C., & Lambert, F. (2020). In vitro and in vivo biocompatibility of calcium-phosphate scaffolds three-dimensional printed by stereolithography for bone regeneration. *J Biomed Mater Res A, 108*(3), 412-425. doi:10.1002/jbm.a.36823

Li, Y., Wu, R., Yu, L., Shen, M., Ding, X., Lu, F., . . . Xu, S. (2021). Rational design of nonstoichiometric bioceramic scaffolds via digital light processing: tuning chemical composition and pore geometry evaluation. *J Biol Eng, 15*(1), 1. doi:10.1186/s13036-020-00252-3

Liang, H., Wang, Y., Chen, S., Liu, Y., Liu, Z., & Bai, J. (2022). Nano-Hydroxyapatite Bone Scaffolds with Different Porous Structures Processed by Digital Light Processing 3D Printing. *Int J Bioprint, 8*(1), 502. doi:10.18063/ijb.v8i1.502

Lim, H. K., Hong, S. J., Byeon, S. J., Chung, S. M., On, S. W., Yang, B. E., . . . Byun, S. H. (2020). 3D-Printed Ceramic Bone Scaffolds with Variable Pore Architectures. *Int J Mol Sci, 21*(18). doi:10.3390/ijms21186942

Luo, Y., Le Fer, G., Dean, D., & Becker, M. L. (2019). 3D Printing of Poly(propylene fumarate) Oligomers: Evaluation of Resin Viscosity, Printing Characteristics and Mechanical Properties. *Biomacromolecules, 20*(4), 1699-1708. doi:10.1021/acs.biomac.9b00076

Luo, Y., Pan, H., Jiang, J., Zhao, C., Zhang, J., Chen, P., . . . Fan, S. (2020). Desktop-Stereolithography 3D Printing of a Polyporous Extracellular Matrix Bioink for Bone Defect Regeneration. *Front Bioeng Biotechnol, 8*, 589094. doi:10.3389/fbioe.2020.589094

Maggi, A., Li, H., & Greer, J. R. (2017). Three-dimensional nano-architected scaffolds with tunable stiffness for efficient bone tissue growth. *Acta Biomater, 63*, 294-305. doi:10.1016/j.actbio.2017.09.007

Mangano, C., Mangano, F., Gobbi, L., Admakin, O., Iketani, S., & Giuliani, A. (2019). Comparative Study between Laser Light Stereo-Lithography 3D-Printed and Traditionally Sintered Biphasic Calcium Phosphate Scaffolds by an Integrated Morphological, Morphometric and Mechanical Analysis. *Int J Mol Sci, 20*(13). doi:10.3390/ijms20133118

Marino, A., Filippeschi, C., Genchi, G. G., Mattoli, V., Mazzolai, B., & Ciofani, G. (2014). The Osteoprint: a bioinspired two-photon polymerized 3-D structure for the enhancement of bone-like cell differentiation. *Acta Biomater, 10*(10), 4304-4313. doi:10.1016/j.actbio.2014.05.032

Miao, S., Zhu, W., Castro, N. J., Nowicki, M., Zhou, X., Cui, H., . . . Zhang, L. G. (2016). 4D printing smart biomedical scaffolds with novel soybean oil epoxidized acrylate. *Sci Rep, 6*, 27226. doi:10.1038/srep27226

Nava, M. M., Di Maggio, N., Zandrini, T., Cerullo, G., Osellame, R., Martin, I., & Raimondi, M. T. (2017). Synthetic niche substrates engineered via two-photon laser polymerization for the expansion of human mesenchymal stromal cells. *J Tissue Eng Regen Med, 11*(10), 2836-2845. doi:10.1002/term.2187

Nettleton, K., Luong, D., Kleinfehn, A. P., Savariau, L., Premanandan, C., & Becker, M. L. (2019). Molecular Mass-Dependent Resorption and Bone Regeneration of 3D Printed PPF Scaffolds in a Critical-Sized Rat Cranial Defect Model. *Adv Healthc Mater, 8*(17), e1900646. doi:10.1002/adhm.201900646

Owen, R., Sherborne, C., Paterson, T., Green, N. H., Reilly, G. C., & Claeyssens, F. (2016). Emulsion templated scaffolds with tunable mechanical properties for bone tissue engineering. *J Mech Behav Biomed Mater, 54*, 159-172. doi:10.1016/j.jmbbm.2015.09.019

Păun, I. A., Mustăciosu, C. C., Popescu, R. C., Călin, B., & Mihăilescu, M. (2020). Collagen/Chitosan Functionalization of Complex 3D Structures Fabricated by Laser Direct Writing via Two-Photon Polymerization for Enhanced Osteogenesis. *Int J Mol Sci, 21*(17). doi:10.3390/ijms21176426

Paun, I. A., Popescu, R. C., Calin, B. S., Mustaciosu, C. C., Dinescu, M., & Luculescu, C. R. (2018). 3D Biomimetic Magnetic Structures for Static Magnetic Field Stimulation of Osteogenesis. *Int J Mol Sci, 19*(2). doi:10.3390/ijms19020495

Paun, I. A., Popescu, R. C., Mustaciosu, C. C., Zamfirescu, M., Calin, B. S., Mihailescu, M., . . . Luculescu, C. R. (2018). Laser-direct writing by two-photon polymerization of 3D honeycomb-like structures for bone regeneration. *Biofabrication, 10*(2), 025009. doi:10.1088/1758-5090/aaa718

Petrochenko, P. E., Torgersen, J., Gruber, P., Hicks, L. A., Zheng, J., Kumar, G., . . . Ovsianikov, A. (2015). Laser 3D printing with sub-microscale resolution of porous elastomeric scaffolds for supporting human bone stem cells. *Adv Healthc Mater, 4*(5), 739-747. doi:10.1002/adhm.201400442

Qin, H., Wei, Y., Han, J., Jiang, X., Yang, X., Wu, Y., . . . Chen, L. (2022). 3D printed bioceramic scaffolds: Adjusting pore dimension is beneficial for mandibular bone defects repair. *J Tissue Eng Regen Med, 16*(4), 409-421. doi:10.1002/term.3287

Schmidleithner, C., Malferarri, S., Palgrave, R., Bomze, D., Schwentenwein, M., & Kalaskar, D. M. (2019). Application of high resolution DLP stereolithography for fabrication of tricalcium phosphate scaffolds for bone regeneration. *Biomed Mater, 14*(4), 045018. doi:10.1088/1748-605X/ab279d

Skoog, S. A., Nguyen, A. K., Kumar, G., Zheng, J., Goering, P. L., Koroleva, A., . . . Narayan, R. J. (2014). Two-photon polymerization of 3-D zirconium oxide hybrid scaffolds for long-term stem cell growth. *Biointerphases, 9*(2), 029014. doi:10.1116/1.4873688

Teotia, A. K., Dienel, K., Qayoom, I., van Bochove, B., Gupta, S., Partanen, J., . . . Kumar, A. (2020). Improved Bone Regeneration in Rabbit Bone Defects Using 3D Printed Composite Scaffolds Functionalized with Osteoinductive Factors. *ACS Appl Mater Interfaces, 12*(43), 48340-48356. doi:10.1021/acsami.0c13851

Tesavibul, P., Chantaweroad, S., Laohaprapanon, A., Channasanon, S., Uppanan, P., Tanodekaew, S., . . . Sitthiseripratip, K. (2015). Biocompatibility of hydroxyapatite scaffolds processed by lithography-based additive manufacturing. *Biomed Mater Eng, 26*(1-2), 31-38. doi:10.3233/bme-151549

Timashev, P., Kuznetsova, D., Koroleva, A., Prodanets, N., Deiwick, A., Piskun, Y., . . . Bagratashvili, V. (2016). Novel biodegradable star-shaped polylactide scaffolds for bone regeneration fabricated by two-photon polymerization. *Nanomedicine (Lond), 11*(9), 1041-1053. doi:10.2217/nnm-2015-0022

Walker, J. M., Bodamer, E., Krebs, O., Luo, Y., Kleinfehn, A., Becker, M. L., & Dean, D. (2017). Effect of Chemical and Physical Properties on the In Vitro Degradation of 3D Printed High Resolution Poly(propylene fumarate) Scaffolds. *Biomacromolecules, 18*(4), 1419-1425. doi:10.1021/acs.biomac.7b00146

Wei, Y., Zhao, D., Cao, Q., Wang, J., Wu, Y., Yuan, B., . . . Zhang, X. (2020). Stereolithography-Based Additive Manufacturing of High-Performance Osteoinductive Calcium Phosphate Ceramics by a Digital Light-Processing System. *ACS Biomater Sci Eng, 6*(3), 1787-1797. doi:10.1021/acsbiomaterials.9b01663

Xu, S., Zhang, H., Li, X., Zhang, X., Liu, H., Xiong, Y., . . . Yu, S. (2022). Fabrication and biological evaluation of porous β-TCP bioceramics produced using digital light processing. *Proc Inst Mech Eng H, 236*(2), 286-294. doi:10.1177/09544119211041186

Yang, Z., Xie, L., Zhang, B., Zhang, G., Huo, F., Zhou, C., . . . Tan, Y. (2022). Preparation of BMP-2/PDA-BCP Bioceramic Scaffold by DLP 3D Printing and its Ability for Inducing Continuous Bone Formation. *Front Bioeng Biotechnol, 10*, 854693. doi:10.3389/fbioe.2022.854693

Zeng, Y., Yan, Y., Yan, H., Liu, C., Li, P., Dong, P., . . . Chen, J. (2018). 3D printing of hydroxyapatite scaffolds withgood mechanical and biocompatible properties by digital light processing. *Journal of Materials Science, 53*(9), 6291-6301. doi:10.1007/s10853-018-1992-2

Zhang, B., Gui, X., Song, P., Xu, X., Guo, L., Han, Y., . . . Zhang, X. (2022). Three-Dimensional Printing of Large-Scale, High-Resolution Bioceramics with Micronano Inner Porosity and Customized Surface Characterization Design for Bone Regeneration. *ACS Appl Mater Interfaces, 14*(7), 8804-8815. doi:10.1021/acsami.1c22868

Zhang, H., Zhang, H., Xiong, Y., Dong, L., & Li, X. (2021). Development of hierarchical porous bioceramic scaffolds with controlled micro/nano surface topography for accelerating bone regeneration. *Mater Sci Eng C Mater Biol Appl, 130*, 112437. doi:10.1016/j.msec.2021.112437

Zhou, X., Castro, N. J., Zhu, W., Cui, H., Aliabouzar, M., Sarkar, K., & Zhang, L. G. (2016). Improved Human Bone Marrow Mesenchymal Stem Cell Osteogenesis in 3D Bioprinted Tissue Scaffolds with Low Intensity Pulsed Ultrasound Stimulation. *Sci Rep, 6*, 32876. doi:10.1038/srep32876

Zhou, X., Zhu, W., Nowicki, M., Miao, S., Cui, H., Holmes, B., . . . Zhang, L. G. (2016). 3D Bioprinting a Cell-Laden Bone Matrix for Breast Cancer Metastasis Study. *ACS Appl Mater Interfaces, 8*(44), 30017-30026. doi:10.1021/acsami.6b10673
